# Chronic obstructive pulmonary disease: update of today’s knowledge and challenges

**DOI:** 10.48101/ujms.v131.15122

**Published:** 2026-10-31

**Authors:** Anders Blomberg

**Affiliations:** Department of Public Health and Clinical Medicine, Umeå University, Umeå, Sweden

This issue of *Upsala Journal of Medical Sciences* discusses chronic obstructive pulmonary disease (COPD) from many important perspectives. It is nowadays well acknowledged that COPD, apart from being characterized by persistent airflow obstruction, is a heterogeneous syndrome including several clinical phenotypes with varying prognosis, treatment responses, and comorbidities (1, 2). The spirometric definition of COPD is under debate: should a fixed ratio vs. lower limit of normal (LLN) or pre- vs. post-bronchodilator values be used, and are relevant spirometric reference values available? Also, COPD prevalence is affected not only by the selected diagnostic criteria but also by a considerable underdiagnosis (or misclassification) globally. While COPD has previously been considered mainly a tobacco smoke-related disease, it is today obvious that many other causes are important in COPD pathogenesis. Furthermore, COPD is increasingly diagnosed in never-smoking individuals; around one-third of middle-aged Swedes with chronic airflow obstruction were found to be never-smokers in the population-based SCAPIS study (Swedish CArdioPulmonary bioImage Study) (3). Thus, the number of clinically relevant phenotypes in COPD has increased the need for better disease characterization to improve prognosis. Here, eosinophilia, lung function decline, comorbidities, and exacerbation frequency are suggested as important contributors.

When it comes to never-smokers with COPD, it is obvious that they show several distinct clinical features such as earlier age of onset, milder airflow obstruction, and more frequently small airways disease, specifically related to a history of asthma. Thus, they constitute a distinct phenotype with unique risk factors and clinical characteristics, yet with a great shortage of knowledge about management and treatment.

Apart from conventional spirometry and computed tomography (CT) scan of the thorax to confirm airway obstruction and emphysema, other modalities are now introduced in the diagnostic arsenal of COPD. Oscillometry and nitrogen washout are found to be sensitive to studying mechanical properties of the lung and ventilation heterogeneity, respectively, whereas airway dimension assessment (AiDA) has been proposed as a means of obtaining information about emphysema. As there are certain gaps in the present knowledge about the development of COPD, these methods may be important as possible tools to diagnose COPD at earlier, preclinical stages. To date, however, a central research question is if and how it is possible to intervene and stop the progression of COPD.

Along with inhaled bronchodilators in the form of long-acting beta-2-agonists and long-acting muscarinic antagonists, corticosteroids (ICS) are included in the treatment arsenal of COPD. While having a number of side effects and being considered a two-edged sword, ICS may have substantial benefits, such as decreased exacerbation risk, lung function improvement, as well as decreased cardiovascular and mortality risks, specifically in patients with high blood eosinophils and a history of exacerbations and/or asthma. On the other hand, ICS should not be used in patients with repeated lung infections and low eosinophil counts. Thus, selecting patients suitable for ICS treatment is important. Here, it should also be emphasized to keep the dose of ICS as low as possible and to thoroughly verify that the patient manages the inhaler therapy. Recently, treatment with biologics has been suggested as an alternative also in COPD patients with high eosinophil numbers and frequent exacerbations (4). However, a biologic should always be added on top and never replace the maintenance inhaler therapy.

Among comorbid conditions in COPD, cardiovascular diseases (CVD) are most important. While they share risk factors in terms of age and smoking history, COPD in itself constitutes an independent risk factor for CVD, along with low physical activity, low socioeconomic class, as well as airflow limitation in itself. Of note, heart diseases are prevalent in both mild and more severe COPD, and cardiovascular mortality is more common than respiratory mortality, especially in mild-to-moderate COPD. The most important processes suggested to contribute to the association between COPD and CVD are lung hyperinflation, hypoxemia, and systemic inflammation. As a COPD exacerbation affects most of these above-mentioned processes negatively, the risk of a cardiovascular event is strongly enhanced after an exacerbation, considerably during the first month but still with a doubled risk up to a year. Given this close connection between COPD and CVD, it is vital to always involve cardiovascular risk factors in the management of COPD, as well as emphasizing relevant COPD medication together with sustained freedom from tobacco, annual influenza vaccination, and the promotion of physical activity.

Dyspnea and fatigue are cardinal symptoms in COPD that reduce daily physical activity, diminish physical capacity and, consequently, adversely affect quality of life. Furthermore, as both physical activity and capacity are strong predictors of mortality, it is vital to increase physical activity in individuals with COPD. Promoting physical activity with interventions is therefore important and has been shown to significantly improve short-term levels of daily physical activity. COPD patients with less than 4,000 steps per day are at risk, and interventions with physical activity should be promoted, employing individualized and behavioral change techniques. Pedometer-guided coaching should be used, as it represents a simple and easy means to assess physical activity. Also, walking is the preferred form of physical activity for most physically inactive individuals with COPD. Exercise training is central to increasing muscular strength and improving cardiovascular function. However, training programs should include both endurance and strength components, as this combination has shown superior outcomes compared with endurance training alone. Pulmonary rehabilitation in COPD is a comprehensive intervention to cover more aspects of the disease and is indicated for all patients with significant symptoms and/or high risk of exacerbation, yet only available to less than 3% of COPD patients globally.

Respiratory insufficiency is a heterogeneous condition based on gas exchange impairment and ventilatory failure, in COPD often modified by comorbidities. Hypoxemia is primarily caused by ventilation–perfusion mismatch, whereas hypercapnia mainly reflects impaired efficiency of alveolar ventilation. In acute COPD exacerbations, controlled oxygen therapy and noninvasive ventilation are the basis for treatment, with a positive impact on both need for intubation and mortality. Oxygen treatment with high-flow nasal cannula may be considered, but as a complement rather than a substitute therapy (5). In chronic respiratory failure, it is important to distinguish between patients with predominant gas exchange impairment and hypoxemia, who would benefit from long-term oxygen therapy, whereas those with ventilatory failure and hypercapnia rather gain from home mechanical ventilation. Of note, obesity hypoventilation syndrome in combination with lung disease (predominantly COPD) is now one of the leading indications for home mechanical ventilation. Thus, when it comes to respiratory insufficiency in COPD, supportive treatment with a phenotype-based and physiology-driven approach should be encouraged. Here, focusing on the leading underlying mechanism, ventilatory failure or gas exchange impairment, along with accounting for comorbidities, such as CVD and obesity, is essential to improve outcomes.

Individuals with COPD often report poor sleep quality in terms of sleep disturbances and insomnia, along with increased daytime sleepiness. Studies also indicate reduced sleep efficiency in COPD, especially in men with COPD. Sleep quality worsens with COPD severity and, overall, about 70% of patients with COPD report difficulty sleeping, i.e. two to three times more common than in the general population. It is suggested that hypoventilation during sleep is more pronounced in COPD and related to other factors such as airflow obstruction. Nocturnal airway symptoms (dyspnea, cough, wheeze, and sputum production) may disturb sleep and, consequently, increase daytime tiredness. Furthermore, as poor sleep also increases the risk of future COPD exacerbations, it is important to improve sleep quality in COPD. In COPD, the most common sleep disorders are insomnia, obstructive sleep apnea (OSA), and restless legs syndrome, all present in approximately 20–30% of patients with COPD. Patients with overlap syndrome (OSA + COPD) have impaired quality of life, increased risk of developing CVD and exacerbations, as well as higher mortality rates. Here, positive airway pressure (PAP) treatment is beneficial to reduce the exacerbation risk.

Taken together, it should be emphasized that COPD is a common diagnosis not only in Sweden but all over the world. COPD is a heterogeneous syndrome including many clinically relevant phenotypes with different prognosis and treatment responses. Furthermore, multimorbidity is common in COPD, with CVD as one of the most important comorbidities when it comes to prognosis. Also, smoking cessation is vital, and physical activity along with pulmonary rehabilitation is the basis for nonpharmacological treatment. While inhalation therapy with bronchodilators and ICS is still the most important medical treatment, biologics are becoming a possible complement.

However, some issues about COPD have to be addressed in the future – researchers and clinicians need to agree on a diagnostic criterion that is clinically relevant. We should also do better to address multimorbidity, develop biomarkers of prognostic value, and, last but not least, fight the great underdiagnosis and misclassification of COPD that still is present worldwide.
